# Hypoxia Induces Renal Epithelial Injury and Activates Fibrotic Signaling Through Up-Regulation of Arginase-II

**DOI:** 10.3389/fphys.2021.773719

**Published:** 2021-11-19

**Authors:** Xiujie Liang, Duilio Michele Potenza, Andrea Brenna, Yiqiong Ma, Zhilong Ren, Xin Cheng, Xiu-Fen Ming, Zhihong Yang

**Affiliations:** Laboratory of Cardiovascular and Aging Research, Department of Endocrinology, Metabolism, and Cardiovascular System, Faculty of Science and Medicine, University of Fribourg, Fribourg, Switzerland

**Keywords:** arginase, collagen, hypoxia, kidney, TGFβ1

## Abstract

The ureohydrolase, type-II arginase (Arg-II), is a mitochondrial enzyme metabolizing L-arginine into urea and L-ornithine and is highly expressed in renal proximal tubular cells (PTC) and upregulated by renal ischemia. Recent studies reported contradictory results on the role of Arg-II in renal injury. The aim of our study is to investigate the function of Arg-II in renal epithelial cell damage under hypoxic conditions. Human renal epithelial cell line HK2 was cultured under hypoxic conditions for 12–48 h. Moreover, *ex vivo* experiments with isolated kidneys from wild-type (WT) and genetic Arg-II deficient mice (*Arg-II^–/–^*) were conducted under normoxic and hypoxic conditions. The results show that hypoxia upregulates Arg-II expression in HK2 cells, which is inhibited by silencing both hypoxia-inducible factors (HIFs) HIF1α and HIF2α. Treatment of the cells with dimethyloxaloylglycine (DMOG) to stabilize HIFα also enhances Arg-II. Interestingly, hypoxia or DMOG upregulates transforming growth factor β1 (TGFβ1) levels and *collagens I*α*1*, which is prevented by *Arg-II* silencing, while TGFβ1-induced *collagen I*α*1* expression is not affected by *Arg-II* silencing. Inhibition of mitochondrial complex-I by rotenone abolishes hypoxia-induced reactive oxygen species (mtROS) and TGFβ1 elevation in the cells. *Ex vivo* experiments show elevated Arg-II and TGFβ1 expression and the injury marker NGAL in the WT mouse kidneys under hypoxic conditions, which is prevented in the *Arg-II^–/–^* mice. Taking together, the results demonstrate that hypoxia activates renal epithelial HIFs-Arg-II-mtROS-TGFβ1-cascade, participating in hypoxia-associated renal injury and fibrosis.

## Introduction

Sufficient supply of oxygen is prerequisite for normal organ function and hypoxia related to many conditions such as high altitude, ischemia, or hypoxemia plays important roles in organ damage and development of diseases including heart disease, renal diseases, neurodegenerative disease, etc. (Lee et al., 2019; Schodel and Ratcliffe, 2019; Burtscher et al., 2021). Hypoxia-inducible factors (HIFs) are central mechanisms regulating cellular adaptation to hypoxia and are transcriptional heterodimers composed of a α-subunit and a β-subunit (Kaelin and Ratcliffe, 2008). While the β-subunit is constitutively expressed, the α-subunits including HIF-1α and HIF-2α are rapidly degraded in the presence of sufficient oxygen (Ivan and Kaelin, 2017). Under normoxia condition, HIF-1α and HIF-2α undergo hydroxylation by prolyl-hydroxylase (PHD), allowing recognition and ubiquitination by von Hippel-Lindau (VHL) and rapid degradation through the E3 ubiquitin ligase complex (Ivan and Kaelin, 2017). Under hypoxic conditions, HIF-1α and HIF-2α escape hydroxylation and degradation and are therefore stabilized and translocated into the nucleus to form heterodimeric complex with the constitutive β-subunit and regulate gene expression, which is critical for cellular adaptation to hypoxic conditions (Ivan and Kaelin, 2017).

Among other organs, kidney is one of the most sensitive organs to hypoxia (Faivre et al., 2020). Renal tissue hypoxia is known to be present in kidney disease and contributes to acute and chronic renal failure (Tanaka et al., 2006; Inoue et al., 2011; Faivre et al., 2020). Proximal tubular cells (PTCs) are highly active cells in the kidney and are responsible for electrolyte and fluid balance, exhibit high metabolic activities and reveal high oxygen demand, and the relatively lower blood supply to this medullary region ensures that the PTCs are particularly sensitive and vulnerable to even modest changes in oxygen supply (Ferenbach and Bonventre, 2015). Studies provide evidence demonstrating an active role of PTCs in pathogenesis of renal diseases, including transition of acute kidney damage to chronic kidney disease manifested by inflammation and tubulointerstitial fibrosis (Canaud and Bonventre, 2015; Gilbert, 2017; Gewin, 2018; Liu et al., 2018; Qi and Yang, 2018; Haraguchi et al., 2020). Besides immune cells, the PTCs are able to produce cytokines that participate in renal inflammation and fibrosis (Yang et al., 2010; Canaud and Bonventre, 2015). Among them, TGFβ1 has been known to be the important player in renal tubulointerstitial fibrosis and can be produced by PTCs in response to insults including hypoxia (Meng et al., 2016; Cho et al., 2019). It has been shown that in the human PTC cell line (HK2), hypoxia causes TGFβ1 release associated with increased production of reactive oxygen species (ROS) from mitochondria and NADPH oxidase 4 (NOX4), leading to renal damage (Cho et al., 2019). In tumor associated fibroblasts, mitochondrial ROS (mtROS) induced by radiation has been reported to enhance TGFβ1 production (Shimura et al., 2018). However, whether mitochondrial dysfunction i.e., mtROS under hypoxic conditions plays a causal role in TGFβ1 production in renal PTCs is not known.

It is interesting to note that the L-arginine-ureohydrolase type-II or arginase type-II (Arg-II) is prominently expressed in the renal PTC straight segment under physiological conditions, but it is also inducible under stress or pathological conditions (Yang and Ming, 2013, 2014; Huang et al., 2021). The enzyme is localized in mitochondria and metabolizes L-arginine to L-ornithine and urea (Wu and Morris, 1998). Upregulation of Arg-II is associated with chronic pro-inflammatory responses and knockout of *Arg-II* generally exerts protective effects in various disease models and in aging (Yepuri et al., 2012; Yang and Ming, 2014; Huang et al., 2021). It is well established that hypoxia is one of the strong stimuli for Arg-II upregulation in various cell types (Krotova et al., 2010; Prieto et al., 2011; Cowburn et al., 2016; Pandey et al., 2018; Liang et al., 2019). It is well known since long time that Arg-II is prominently expressed in the kidney (Miyanaka et al., 1998; Levillain et al., 2005; Choi et al., 2012). Investigation of the function of Arg-II in renal physiology and pathophysiology are just emerging in recent years and contradictory results have been reported in the literature (Huang et al., 2018, 2021; Ansermet et al., 2020; Hara et al., 2020). Both protective and detrimental effects of Arg-II in renal damage are reported in mouse models in response to ischemia (Ansermet et al., 2020; Hara et al., 2020). Taken into account that the mitochondrial Arg-II and TGFβ1 are upregulated by hypoxia (Krotova et al., 2010; Prieto et al., 2011; Cowburn et al., 2016; Pandey et al., 2018; Liang et al., 2019) and over-expression of Arg-II in the HK2 cells causes mtROS generation and enhances TGFβ1 production (Huang et al., 2021), we hypothesize that hypoxia causes renal tubular cell damage through up-regulation of Arg-II-mtROS-TGFβ1 cascade.

## Materials and Methods

### Reagents

Reagents were obtained or purchased from the following sources: rabbit antibody against Arg-II (#55003) was from Cell Signaling Technology (Danvers, United States); mouse antibody against HIF1α (610958) was from BD Biosciences (New Jersey, United States); rabbit antibody against HIF2α (PAB12124) was from Abnova (Taipei, Taiwan); mouse antibody against tubulin (T5168), mouse antibody against β-actin (A5441), dimethyloxaloylglycine (DMOG), and rotenone were from Sigma-Aldrich (St. Louis, Missouri, United States); Rabbit anti-NGAL (ab63929) and rabbit antibody against TGFβ1 (ab215715) were from Abcam (Cambridge, United Kingdom); Alexa Fluor 488-conjugated goat anti-rabbit IgG (H + L) secondary Ab (A-11008), MitoSox (M36008) were from Thermo Fisher Scientific (Waltham, MA, United States). Secondary Alexa fluor 680 conjugated goat anti-mouse IgG (A21057) and DAPI (4′,6-Diamidino-2-Phenylindole, Dihydrochloride) (D1306) were from Invitrogen (Lucerne, Switzerland); IRDye 800-conjugated affinity purified goad anti-rabbit IgG (926-32211) was from BioConcept (Alschwil, Switzerland); All other materials and cell culture media were from Gibco/Thermo Fisher Scientific (Waltham, MA United States).

### Recombinant Adenovirus

The Recombinant adenovirus (rAd) expressing shRNA targeting human Arg-II driven by the U6 promoter (rAd/U6-hArg-II^shRNA^) and control rAd expressing shRNA targeting LacZ (rAd/U6-LacZ^shRNA^) were generated as described previously (Ming et al., 2009). Generation of rAd expressing shRNA targeting human HIF1α and HIF2α driven by the U6 promoter (rAd/U6-hHIF1α^shRNA^ and rAd/U6-hHIF2α^shRNA^, respectively) was carried out with the Gateway Technology. The targeting sequences are (only the sense strand is shown): **GCCGAGGAAGAACTATGAACA** for human HIF1α; **CGA CCTGAAGATTGAAGTGAT** for human HIF2α.

### Cell Culture and Adenoviral Transduction of the Cells

The HK-2 cells (a human proximal tubular epithelial cell line) were purchased from American Type Culture Collection (ATCC, Manassas, VA, United States) and cultured in Dulbecco modified Eagle medium/F12 (DMEM/F12) supplemented with 10% FBS, 100 U/mL penicillin, and 100 μg/mL streptomycin. The cells were maintained at 37°C in a humidified incubator containing a 5% CO2 atmosphere. To silence Arg-II, HIF1α, or HIF2α alone, the cells were seeded at the 6-cm dish for 24 h and transduced first with the rAd at titers of 100 Multiplicity of Infection (MOI) and cultured in complete medium for 2 days and then in serum-free medium for another 24 h before experiments. To silence both HIF1α and HIF2α, the cells were seeded at the 6-cm dish for 24 h and transduced first with the rAd-sh-HIF1α at titers of 100 MOI and cultured in complete medium for 1 day, and then transduced with the rAd-sh-HIF2α for 2 days followed by serum-free starvation for 24 h before experiments. Hypoxic conditions were achieved by placing the cultured cells in a Coy *In Vitro* Hypoxic Cabinet System (The Coy Laboratory Products, Grass Lake, MI United States) at 1% O_2_ with premixed gas of 5% CO_2_/95% N_2_.

### Immunoblotting

Cell lysate preparation, SDS-PAGE and immunoblotting, antibody incubation, and signal detection were conducted as previously described (Ming et al., 2012). Cell or kidney tissue extracts were prepared by lysing cells or tissue powders on ice for 15 min in the lysis buffer containing the following contents (mmol/L): 20 Tris.HCl, 138 NaCl, 2.7 KCl with pH 8.0, 1 MgCl_2_, 1 CaCl_2_, 1 sodium-o-vanadate, 0.02 leupeptin, 0.018 pepstatin, 5 EDTA and 20 NaF supplemented with 5% glycerol, 1% NP-40. Cell/tissue debris and nuclei were removed by centrifugation at 10,000 × g for 10 min at 4°C. Protein concentration was determined with Bio-Rad DCTM Protein Assay Kit according to the manufacturer’s instruction. 40 μg extracts were subjected to SDS-PAGE and transferred to an Immobilon-P membrane (Millipore). The membrane was immersed in 100% methanol followed by washing with PBST buffer and then incubated overnight with the corresponding primary antibody at 4°C with gentle agitation overnight after blocking with 5% skimmed milk. The membrane was then incubated with either anti-mouse (Alexa fluor 680 conjugated) or anti-rabbit (IRDye 800 conjugated) secondary antibodies for 2 h. After three times of washing in PBST buffer, signals on the membrane were visualized using Odyssey Infrared Imaging System (LI-COR Biosciences). Quantification of the signals was performed using Li-Cor Image Studio Software. The information of antibodies used for immunoblotting was presented in Supplementary Table 1.

### Quantitative Real-Time Reverse Transcription Polymerase Chain Reaction

Total RNA extraction and mRNA expression analysis by 2-step quantitative real-time reverse-transcription polymerase chain reaction (qRT-PCR) were performed as described previously (Ming et al., 2012). The mRNA expression levels of all genes were normalized to the reference gene, succinate dehydrogenase complex flavoprotein subunit A (*sdha)* (for hypoxia experiments) or glyceraldehyde-3-phosphate dehydrogenase (*gapdh*) for TGFβ1 stimulation), due to the fact that TGFβ1 altered sdha but not gapdh (Supplementary Figure 1). The primer sequences of human genes are as follow:

*tgf*β*1*-F: 5′-CCC AGC ATC TGC AAA GCT C-3′
*tgf*β*1*-R: 5′-GTC AAT GTA CAG CTG CCG CA-3′
*il-1*β-F: 5′-TCT TCG ACA CAT GGG ATA ACG-3′
*il-1*β-R: 5′-TCC CGG AGC GTG CAG TT-3′
*mcp-1*-F: 5′-GAT CTC AGT GCA GAG GCT CG-3′
*mcp-1*-R: 5′-TGC TTG TCC AGG TGG TCC AT-3′
*tnf*α-F: 5′-CCC AGG GAC CTC TCT CTA ATC A-3′
*tnf*α-R: 5′-GCT ACA GGC TTG TCA CTC GG-3′
*collagen 1*α*1*-F: 5′-GTT CGT GAC CGT GAC CTC G-3′
*collagen 1*α*1*-R: 5′-TCT TGT CCT TGG GGT TCT TGC-3′
*sdha*-F: 5′-TGG GAA CAA GAG GGC ATC-3′
*sdha*-R: 5′-CCA CCA CTG CAT CAA ATT CAT-3′
*gapdh-F:* 5′-TGCACCACCAACTGCTTAGC-3′
*gapdh-R*: 5′-GGCATGGACTGTGGTCATGAG-3′

### Mitochondrial Superoxide Detection (MitoSox Staining)

Mitochondrial superoxide generation was studied using MitoSox (Huang et al., 2021). The cells were incubated with MitoSox at the concentration of 5 μmol/L for 10 min. After washing, the cells were then fixed with 3.7% of paraformaldehyde followed by counterstaining with DAPI and then subjected to imaging under the Leica TCS SP5 confocal laser microscope. To study mitochondrial reactive oxygen species (ROS) generation, some cells were treated with rotenone (2 μmol/L, 1 h) followed by subjection to MitoSox as above described.

### *Ex vivo* Experiments With Isolated Mouse Kidneys

*Arg-II^–/–^* mice were kindly provided by Dr. William O’Brien (Shi et al., 2001) and backcrossed to C57BL/6 J for more than 10 generations. Genotypes of mice were confirmed by polymerase chain reaction (PCR) as previously described (Shi et al., 2001). Offspring of WT and *arg-II^–/–^* mice were generated by interbred from hetero/hetero cross. Mice were housed at 23°C with 12-hlight-dark cycle. Animals were fed a normal chow diet and have free access to water. WT and *Arg-II^–/–^* male mice at age of 22 months (three in each group) were anesthesized and sacrificed by exsanguision. Kidneys from the WT and *Arg-II^–/–^* mice were quickly excised and cut into two half horizontally for *ex vivo* experiments. Kidney tissues were placed into a 6-well plate and immersed in RPMI-1640 medium supplemented with insulin-transferrin-selenium (ITS) and penicillin/streptomicin (1%). The amount of medium was just enough to cover the tissues. The tissues were incubated in a Coy *In Vitro* Hypoxic Cabinet System (The Coy Laboratory Products, Grass Lake, MI, United States) at 1% O_2_ with premixed gas of 5% CO_2_/95% N_2_ or in normoxic chambers as controls for 24 h. The kidney tissues were then fixed with 3.7% paraformaldehyde, and then embedded in paraffin for immunofluorescence staining experiments. Experimental work with animals was approved by the Ethical Committee of Veterinary Office of Fribourg Switzerland (2018_01_FR) and performed in compliance with guidelines on animal experimentation at our institution.

### Immunofluorescence Staining

After *ex vivo* exposure to normoxia/hypoxia conditions, kidneys were fixed with 3.7% paraformaldehyde and embedded in paraffin. Horizontal central transverse sections through the middle of the kidney (5 μm) were prepared with Microtome. After deparaffinization in xylene (2 times, 10 min for each), the sections were treated in ethanol (twice in 100% ethanol, twice in 95% ethanol, and once in 80% ethanol for 3 min, sequentially) followed by antigen retrieval (Tris- EDTA buffer, pH 9.0 for Arg-II, and NGAL; citrate buffer, pH 6.0 for TGFβ1) in a pressure cooker. For immunofluorescence staining, the transverse sections (5 μm) were blocked with 1% BSA and 10% goat serum for 1 h and incubated with primary antibodies at 4 °C overnight and subsequently with Alexa Fluor 488–conjugated goat anti-rabbit IgG (H + L) or goat anti-mouse IgG (H + L) for 2 h at room temperature in darkness followed by counterstaining with 300 nmol/L DAPI for 5 min. Negative controls were performed by omitting the primary antibodies (Supplementary Figure 2). The information of antibodies used for immunofluorescence staining was presented in Supplementary Table 1.

### Statistical Analysis

In all experiments, n indicates the number of independent experiments or animals. The Kolmogorov–Smirnov test was used to first determine whether the data deviate from Gaussian distributions. Since all data are normally distributed, statistical analysis was performed with the Student’s *t*-test for unpaired observations or ANOVA with Bonferroni’s post-test, and data are expressed as mean ± SD. Differences were considered statistically significant at *P* < 0.05.

## Results

### Hypoxia Enhances Arg-II Levels Through HIF1α or HIF2α in HK-2 Cells

**FIGURE 1:**
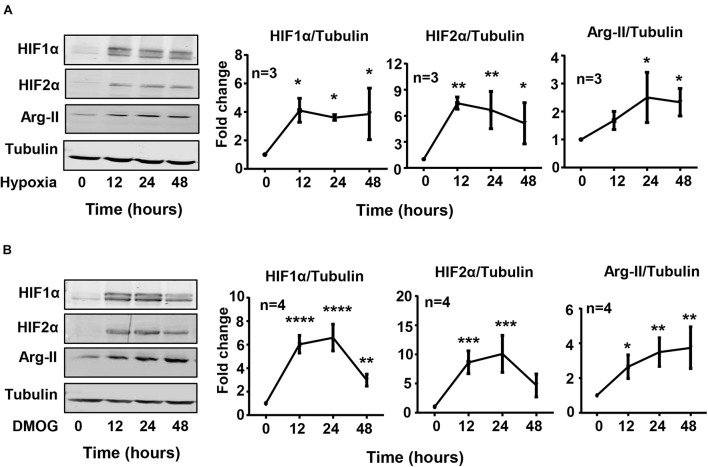
Hypoxia and DMOG up-regulate Arg-II in HK-2 cells. **(A)** HK-2 cells were cultured under hypoxic condition (1% O_2_) for indicated time. Immunoblotting reveals that hypoxia increases the protein level of HIF1α, HIF2α, and Arg-II in HK-2 cells. **(B)** Effects of DMOG (1 mmol/L) on HIF1α, HIF2α, and Arg-II. The graphs on the right show the quantification of the signals on immunoblots. Data are presented as mean ± SD. *P < 0.05, **P < 0.01, ***P < 0.001, ****P < 0.0001 vs. control (time 0, normoxia). ANOVA with Bonferroni *post hoc* test was performed.

**FIGURE 2:**
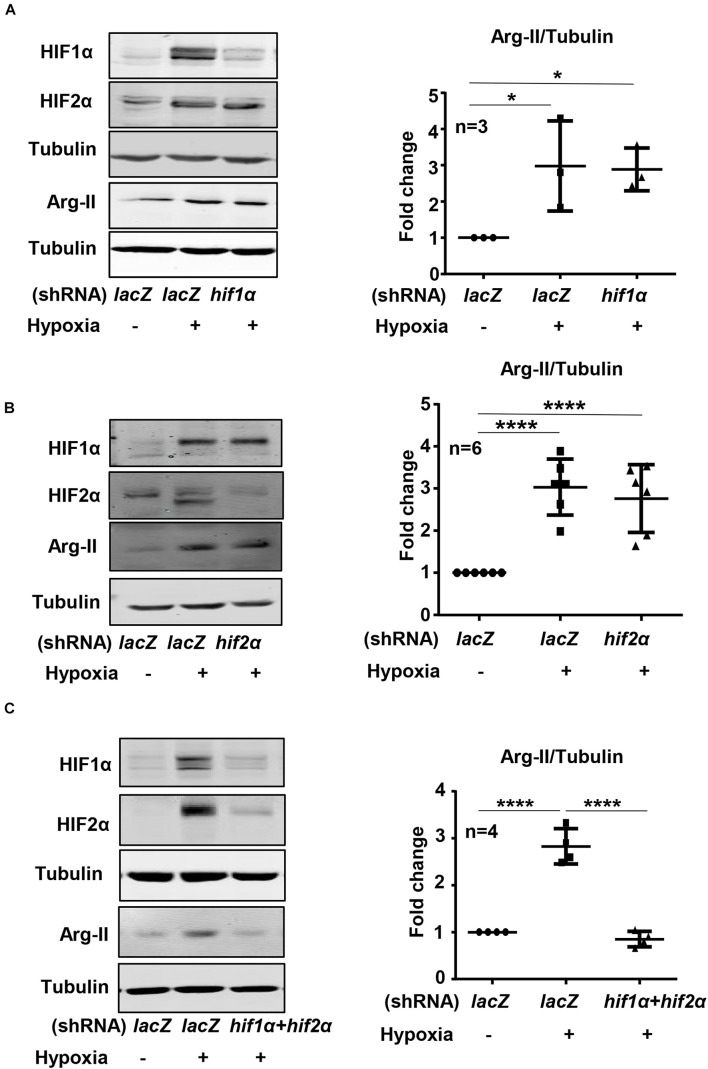
Effects of HIF-silencing on Arg-II expression under hypoxia conditions in HK-2 cells. HK-2 cells were transduced with rAd/U6-LacZ^shRNA^ as control or rAd/U6-*hif1*α^shRNA^ and rAd/U6-*hif2*α^shRNA^ to silence *hif1*α and hif2α gene, respectively. Immunoblotting analyses illustrating effects of **(A)** *hif1*α silencing, **(B)** *hif2*α silencing, and **(C)** simultaneous silencing of both *hif1*α and *hif2*α on Arg-II upregulation in HK-2 cells under hypoxia conditions. The graphs on the right indicate the quantification of the signals on the immunoblots. Data are presented as mean ± SD. *P < 0.05, ****P < 0.0001 between the indicated groups. ANOVA with Bonferroni *post hoc* test was performed.

Exposure of HK-2 cells to hypoxic condition (1% O_2_) over 48 h enhanced HIF1α and HIF2α levels with concomitant increase in Arg-II levels in a time-dependent manner (Figure 1A). The expression of Arg-II in the cells was significantly increased after 24 h of hypoxia exposure and maintained high over the time period (Figure 1A). Treatment of the cells with dimethyloxalylglycine (DMOG), an inhibitor of the prolyl-4-hydroxylase (PHD), to stabilize HIFs, also elevated Arg-II levels (Figure 1B). The results suggest that hypoxia may enhance Arg-II through HIFs. To confirm this hypothesis, HIF1α or HIF2α or both HIFs were silenced with rAd-mediated shRNA and the cells were then exposed to either normoxic or hypoxic conditions for 48 h. The specificity and efficiency of HIF1α and HIF2α silencing were confirmed by immunoblotting (Figures 2A–C). Of note, silencing either *hif1*α or *hif2*α alone was not able to significantly alter Arg-II levels in response to hypoxia (Figures 2A,B), whereas simultaneous silencing of both *hif1*α and *hif2*α prevented hypoxia-induced Arg-II upregulation (Figure 2C). The results demonstrate that either HIF1α or HIF2α is sufficient to mediate hypoxia-induced Arg-II in the renal epithelial cells.

### Effects of Hypoxia on Cytokine/Chemokine Expression in HK-2 Cells

**FIGURE 3:**
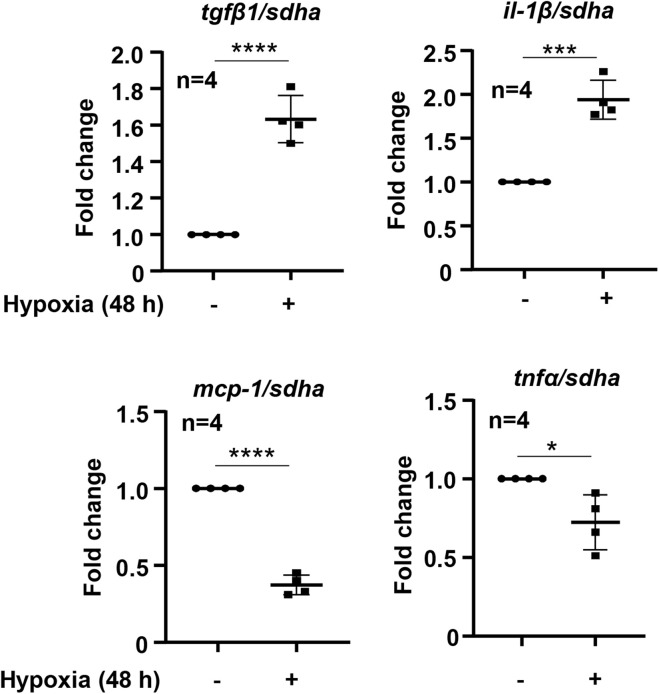
Effects of hypoxia on cytokine expression in HK-2 cells. HK-2 cells were cultured under normoxia or hypoxia conditions for 48 h. The mRNA levels of *tgf*β*1, il-1*β, mcp-1, and *tnf*α were analyzed by qRT-PCR. Data are showed as mean ± SD. *P < 0.05, ***P < 0.001, ****P < 0.0001 between the indicated groups. Un-paired Student’s *t*-test was performed.

**FIGURE 4:**
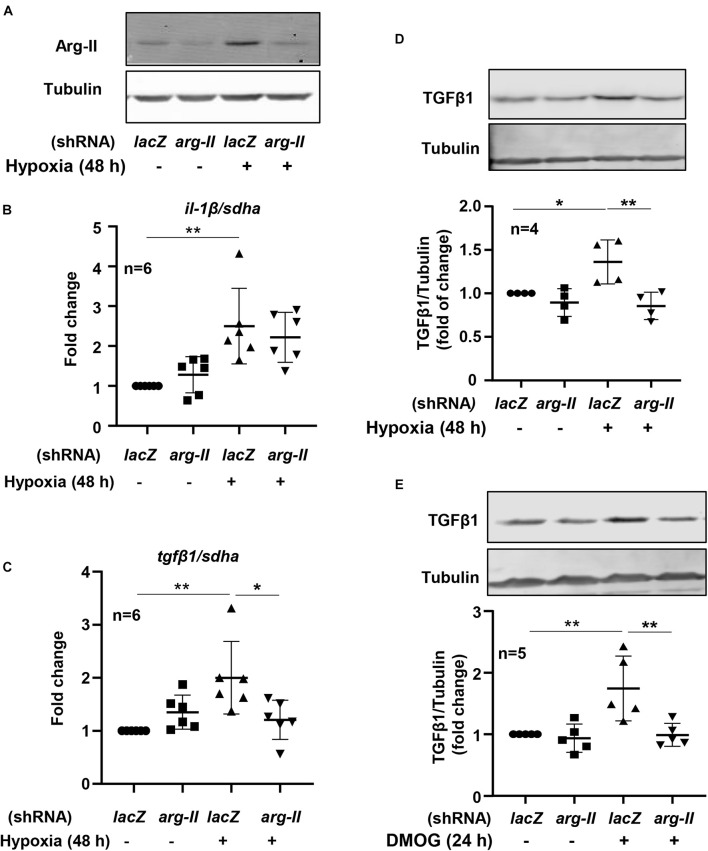
Arg-II mediates hypoxia-induced TGFβ1 upregulation in HK-2 cells. The cells were transduced with rAd/U6-*lacZ*^shRNA^ as control or rAd/U6-*arg-II*^shRNA^ to silence *arg-II* gene, and then incubated under normoxia or hypoxia conditions for 48 h or treated with or without DMOG (1 mmol/L) for 24 h. **(A)** Immunoblotting shows efficiency of *arg-II* silencing; **(B)** qRT-PCR shows effects of *arg-II* silencing on *il-1*β and **(C)** on *tgf*β*1* expression; **(D)** immunoblotting analysis on TGFβ1 protein levels under hypoxia conditions and **(E)** in cells treated with DMOG. *P < 0.05, **P < 0.01 between the indicated groups. ANOVA with Bonferroni *post hoc* test was performed.

To study the effects of hypoxia on renal tubular epithelial cells, HK-2 cells were exposed to hypoxic or normoxic conditions for 48 h. As shown in the Figure 3, hypoxia significantly enhanced *tgf*β*1* and *il-1*β expression but decreased *mcp1* and *tnf*α expression (Figure 3). We therefore focused on whether Arg-II is involved in regulation of *tgf*β*1* and *il-1*β expression under hypoxic condition in the HK-2 cells. Silencing *Arg-II* prevented hypoxia-induced Arg-II upregulation as confirmed by immunoblotting (Figure 4A). *Arg-II* silencing did not show significant effects on *il-1*β (Figure 4B), but prevented hypoxia-induced increase in *tgf*β*1* mRNA expression (Figure 4C) and protein levels (Figure 4D). Furthermore, TGFβ1 protein levels were enhanced in cells treated with DMOG, which was also prevented by silencing Arg-II (Figure 4E), demonstrating that HIF-Arg-II pathway is responsible for elevated TGFβ1 levels under hypoxic conditions.

### Hypoxia Enhances *Collagen* Expression via Arg-II-TGFβ1

**FIGURE 5:**
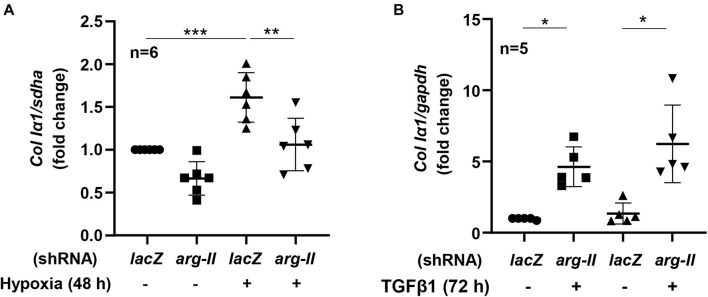
Effects of Arg-II silencing and TGFβ1 on *collagen I*α*1* expression in HK2 cells. The cells were exposed either to **(A)** hypoxia (48 h) or stimulated with **(B)** TGFβ1 (5 ng/mL, 72 h) and *collagen Ia1 gene (Col Ia1)* was analyzed. Data are shown as mean ± SD. *P < 0.05, **p < 0.01, and ***P < 0.001 between the indicated groups. ANOVA with Bonferroni *post hoc* test was performed.

HK-2 cells exposed to hypoxia for 48 h had increased *collagen Ia1* expression, which was inhibited by *Arg-II* silencing (Figure 5A). Moreover, treatment of TGFβ1 (5 ng/mL, 72 h) indeed enhanced expression of *colI*α*1* in the cells, which was not affected by *Arg-II* silencing (Figure 5B). The results demonstrate that Arg-II is upstream of TGFβ1 under hypoxic conditions.

### Roles of mtROS in Arg-II-Elevated TGFβ1 Expression Under Hypoxic Conditions

**FIGURE 6:**
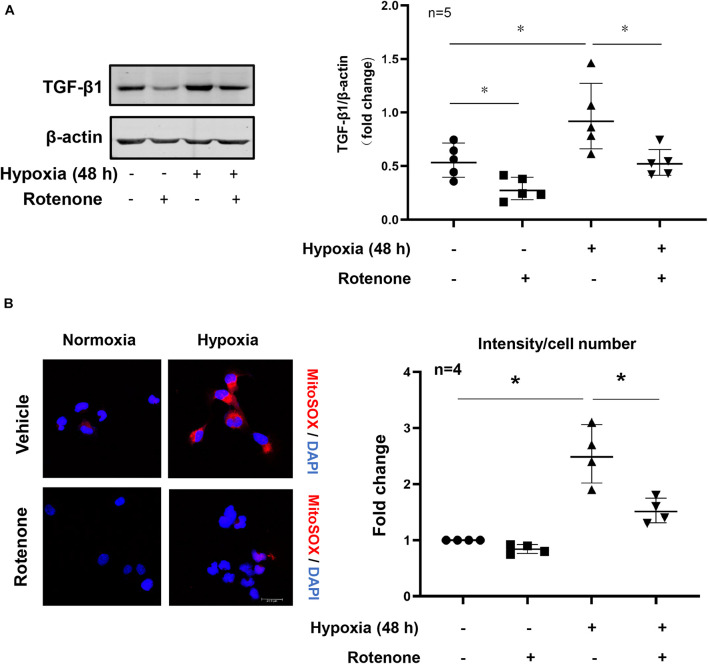
Effects of mitochondrial stress on hypoxia-induced TGFβ1 expression in HK2 cells. **(A)** Immunoblotting analysis of TGFβ1 expression in HK2 cells which were pretreated with rotenone (2 μmol/L for 1 h) and then exposed to hypoxia conditions for 48 h; the graph of right shows quantification of the signals on immunoblots; **(B)** cellular mitochondrial ROS generation as detected by MitoSox staining (red) followed by nuclei staining with DAPI under confocal microscope. The plot graph shows quantification of the signals. Scale bar = 29.9 μm. *p < 0.05 between the indicated groups. Student’s *t*-test was performed between the indicated two groups.

Under hypoxia conditions, cellular TGFβ1 production was enhanced as demonstrated by immunoblotting, which was inhibited by the mitochondrial complex-I inhibitor rotenone (Figure 6A). In parallel, the inhibitor also abolished the increase in cellular production of mtROS as demonstrated by MitoSox signals examined under immunofluorescence confocal microscopy (Figure 6B). The results show that hypoxia enhances mitochondrial ROS generation which mediates hypoxia-induced TGFβ1 production in the renal epithelial cells.

### Hypoxia Enhances Tubular Damage and TGFβ1 Expression in Renal PTCs in *ex vivo* Kidney Culture Models: Inhibition by *Arg-II* Knockout

**FIGURE 7:**
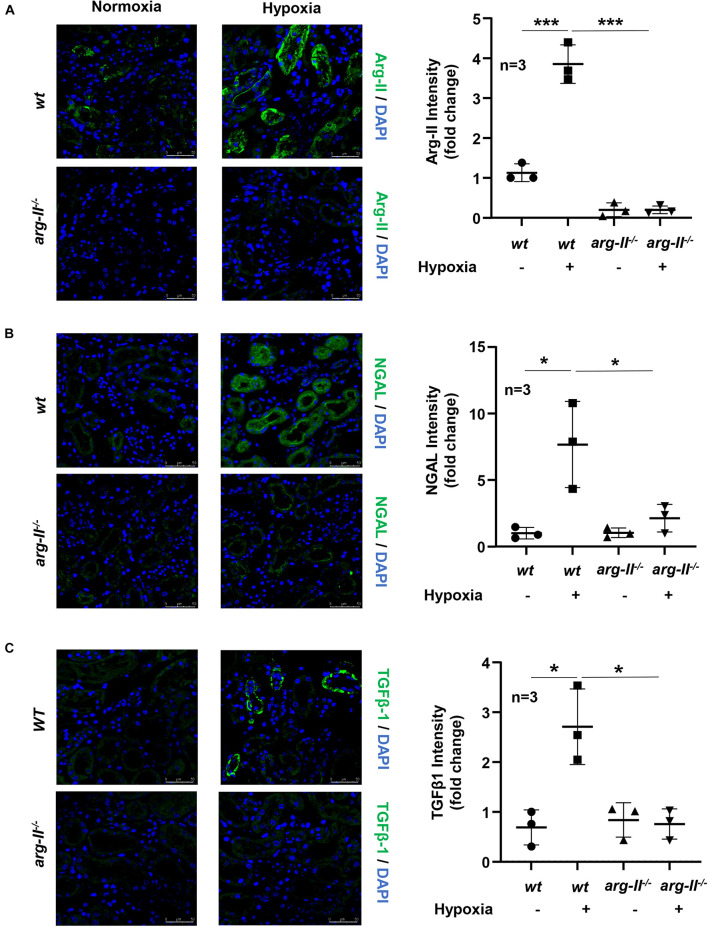
Effects of Arg-II deficiency on hypoxia-induced damage in kidneys. Confocal immunofluorescence staining of **(A)** Arg-II, **(B)** NGAL and **(C)** TGFβ1 and DAPI (blue) in *ex vivo* isolated kidneys of *wt* and *arg-II^–/–^* mice exposed to normoxia and hypoxia conditions (1%, 24 h). Representative images of merged images are shown (*n* = 3 animals per group). Quantifications of fluorescence intensity signals are shown in the bar graphs on the right. *p < 0.05, ***p < 0.001 between the indicated groups. Scale bar = 50 μm. ANOVA with Bonferroni *post hoc* test was performed.

*Ex vivo* experiments with isolated kidney tissues exposed to hypoxic conditions for 24 h were performed. This experiment approach avoids effects of systemic knockout of Arg-II on kidney. In line with the results obtained in cultured HK2 cells, Arg-II in kidney exposed to hypoxia was elevated as compared to the normoxic conditions (Figure 7A). An enhanced epithelial injury as analyzed by epithelial injury marker NGAL (Figure 7B) and an elevated TGFβ1 staining in the renal epithelial cells were observed in the hypoxic kidneys (Figure 7C). These effects of hypoxia were markedly reduced in the *Arg-II^–/–^* mouse kidneys (Figures 7B,C).

## Discussion

It is known that the renal PTCs are very sensitive and susceptible to insults, including hypoxia, due to their high metabolic activity and therefore high oxygen demand (Ferenbach and Bonventre, 2015). Compelling evidence demonstrates that PTCs play a key role in the pathogenesis of the kidney diseases, including acute kidney diseases and chronic kidney diseases (Qi and Yang, 2018; Gerhardt et al., 2021). Indeed, using cultured human renal epithelial cell line HK-2 or *ex vivo* experiments with isolated kidney tissues in culture exposed to hypoxic conditions, we demonstrate that hypoxia causes renal epithelial injury as demonstrated by elevated NGAL levels, the epithelial injury marker, accompanied by upregulation of *collagen Ia1*, TGFβ1, and Arg-II levels. These detrimental effects of hypoxia are abolished by silencing or ablation of *Arg-II* in the cells or in the kidneys of *Arg-II^–/–^* mice, respectively. The results demonstrate a critical role of epithelial Arg-II in hypoxia-induced renal epithelial damage and activation of fibrotic signaling cascade.

Arg-II is a mitochondrial enzyme and is exclusively and highly expressed in the renal PTC straight segment (Huang et al., 2021). Emerging evidence indicates that upregulation of Arg-II plays a detrimental role in kidney of mouse models of ischemia and diabetes (Morris et al., 2011; Toque et al., 2013; You et al., 2013). Indeed, pharmacological inhibition and genetic deficiency of *Arg-II* have been shown to decrease renal injuries in various diabetic mouse models (Morris et al., 2011; You et al., 2013). Various factors are able to upregulate Arg-II in different cell types or under pathological conditions (Yang and Ming, 2014). One of the most potent stimuli of Arg-II upregulation is hypoxia. Similar to vascular cells (Liang et al., 2019), Arg-II is strongly upregulated in response to hypoxia in the renal epithelial cells as demonstrated in the present study either in cultured cell model or in *ex vivo* models of isolated kidney. Of note, either HIF1α or HIF2α can mediate hypoxia-induced upregulation of Arg-II, since silencing either one does not affect Arg-II level, while simultaneous silencing of both HIFs prevents Arg-II upregulation under hypoxia condition. This finding is slightly different from previous studies showing that the hypoxia-induced upregulation of Arg-II is mediated through HIF1α in human umbilical vein endothelial cells (Liang et al., 2019) and HIF2α in pulmonary endothelial cells (Krotova et al., 2010). This discrepancy of Arg-II upregulation by HIFs is likely cell-type specific. The role of HIFs in upregulating Arg-II is further supported by the fact that stabilization of HIFs by the PHD inhibitor DMOG mimicked the effect of hypoxia. Although studies show that HIF1α is induced in tubular cells under hypoxia condition in the kidney by different hypoxic stimuli (Rosenberger et al., 2002) and the degree of hypoxia is well correlated with upregulation of HIF-regulated genes and tubulointerstitial injury (Tanaka et al., 2006), which is an inevitable outcome in chronic kidney disease, the role of HIFs in renal diseases are disputed and may depend on disease conditions. For example, it has been shown that overexpression of HIF1α promotes and inhibition of it attenuates the progression of renal fibrosis in kidney disease models (Nayak et al., 2016; Kabei et al., 2018; Bessho et al., 2019). On the other hand, deficiency of HIF1α in mice accelerated diabetic kidney disease progression (Jiao et al., 2018) and stabilization of HIF1α by PHD inhibitor attenuates ischemic kidney injury (Nordquist et al., 2015). This may be due to the multiple functions or regulation of multiple downstream targets of HIFs. Since Arg-II lies downstream of HIFs in response to hypoxia, targeting Arg-II would achieve more specific effects in protection against kidney damage induced by hypoxia resulting from various pathological conditions.

Hypoxia plays a critical role in development of chronic kidney disease (Faivre et al., 2020). However, the underlying mechanisms are not fully understood. Our present study showed that hypoxia causes renal epithelial damage as demonstrated by enhanced injury marker NGAL in the kidneys exposed to hypoxia. It has been shown that hypoxia induces renal epithelial injury and is able to stimulate the PTCs to produce matrix (Duffield, 2014). The PTCs, besides the active functions in reabsorption of glomerular-filtrated substances, are able to produce pro-inflammatory and pro-fibrotic factors upon injury (Higgins et al., 2007; Huang et al., 2021). In the present study, we demonstrate that hypoxia promotes expression of cytokines such as IL-1β and TGFβ1, the latter is dependent on Arg-II, since silencing *Arg-II* in the cells or *Arg-II^–/–^* deficiency in mouse kidneys prevents hypoxia-induced increase in TGFβ1 levels. The data suggest that TGFβ1 lies down-stream of Arg-II under hypoxic conditions. TGFβ1 is the master player in tissue fibrosis including renal fibrosis (Meng et al., 2016). Previous studies also showed that TGFβ1 could be induced in PTCs in CKD and in aging kidney (Chung et al., 2018; Huang et al., 2021), which plays a role in renal fibrosis. In the present study, we further demonstrate that hypoxia induces TGFβ1 expression in epithelial cells, resulting in activation of pro-fibrotic signaling such as production of collagen. Importantly, the hypoxia-induced stimulation of TGFβ1 and collagen expression as well as the injury marker NGAL in the epithelial cells could be inhibited by *Arg-II* silencing or in *Arg-II^–/–^* mouse kidneys. The fact that TGFβ1-induced expression of *collagen* is not influenced by *Arg-II* silencing in cultured HK2 cells further supports our conclusion that TGFβ1 lies down-stream of Arg-II under hypoxia condition. Shved et al. (2017) analyzed gene expression profiles in HK2 cells in response to hypoxia conditions. Data extraction from their database shows augmented expression of several collagen genes and majority of the collagen gene induction are prevented by knockout of either HIF1α or HIF2α or both together (Supplementary Figure 3). The findings are in line with our results in the present study.

It is well known that hypoxia causes mitochondrial dysfunction, leading to enhanced mtROS generation and cellular damage and ultimately renal disease (Honda et al., 2019). We further investigated whether mtROS production plays a role in hypoxia-stimulated TGFβ1 expression in the tubular cells. Indeed, treatment of the cells with the mitochondrial complex-I inhibitors, rotenone, which inhibits mtROS generation, is capable of preventing the increase in TGFβ1 expression induced by hypoxia. The results demonstrate that mitochondrial dysfunction, i.e., mtROS generation, is mediating the upregulation of TGFβ1 production by hypoxia in renal epithelial cells. Our results are in line with the findings by Zhang et al. (2018) showing that inhibition of mtROS by rotenone suppresses renal inflammation and fibrosis in the ischemic kidney injury model. We would like to point out at this stage that our hypoxic model does not involve the aspect of reperfusion injury which occurs *in vivo* during intervention and contributed by complex cellular and molecular mechanisms (Agarwal et al., 2016). Nevertheless, the results of our study explored a role of Arg-II-mtROS-TGFb1 in hypoxia-induced PTC damage and is line with findings of the study published by Hara et al. (2020).

## Conclusion

**FIGURE 8:**
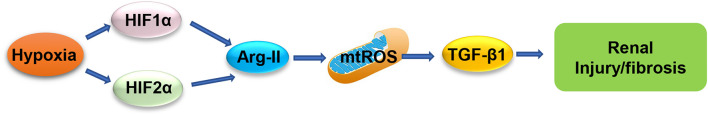
Schematic illustration of mechanisms of hypoxia-induced renal epithelial injury and fibrotic pathway through HIFs-Arg-II-mtROS-TGFβ1 signaling.

In conclusion, our study implicates that hypoxia induces the HIF-Arg-II-mtROS-TGFβ1 cascade in renal PTCs, resulting in renal epithelial injury and contributing to fibrotic process (Figure 8). The results suggest that targeting Arg-II may be beneficial in prevention or treatment of various kidney disease linked to hypoxia conditions.

## Data Availability Statement

The original contributions presented in the study are included in the article/Supplementary Material, further inquiries can be directed to the corresponding author/s.

## Ethics Statement

The animal study was reviewed and approved by the Ethical Committee of Veterinary Office of Fribourg Switzerland.

## Author Contributions

XL, DP, AB, YM, ZR, and XC: acquisition, data analysis, interpretation of data for the work, preparation of figures and drafted the manuscript for important intellectual content. X-FM and ZY: design the work, analyzed and interpreted the research concept, and drafted the manuscript for important intellectual content. All authors agree to be accountable for the content of the work.

## Conflict of Interest

The authors declare that the research was conducted in the absence of any commercial or financial relationships that could be construed as a potential conflict of interest.

## Publisher’s Note

All claims expressed in this article are solely those of the authors and do not necessarily represent those of their affiliated organizations, or those of the publisher, the editors and the reviewers. Any product that may be evaluated in this article, or claim that may be made by its manufacturer, is not guaranteed or endorsed by the publisher.
