# Supplementary material for: Hypoxia Induces Renal Epithelial Injury and Activates Fibrotic Signaling Through Up-Regulation of Arginase-II
Source: Front Physiol. 2021 Nov 19;12:773719. doi: 10.3389/fphys.2021.773719 (PMC8640467; doi:10.3389/fphys.2021.773719)
Supplement: Supplementary file 1 [file Presentation_1.PPTX]

## Slide 1
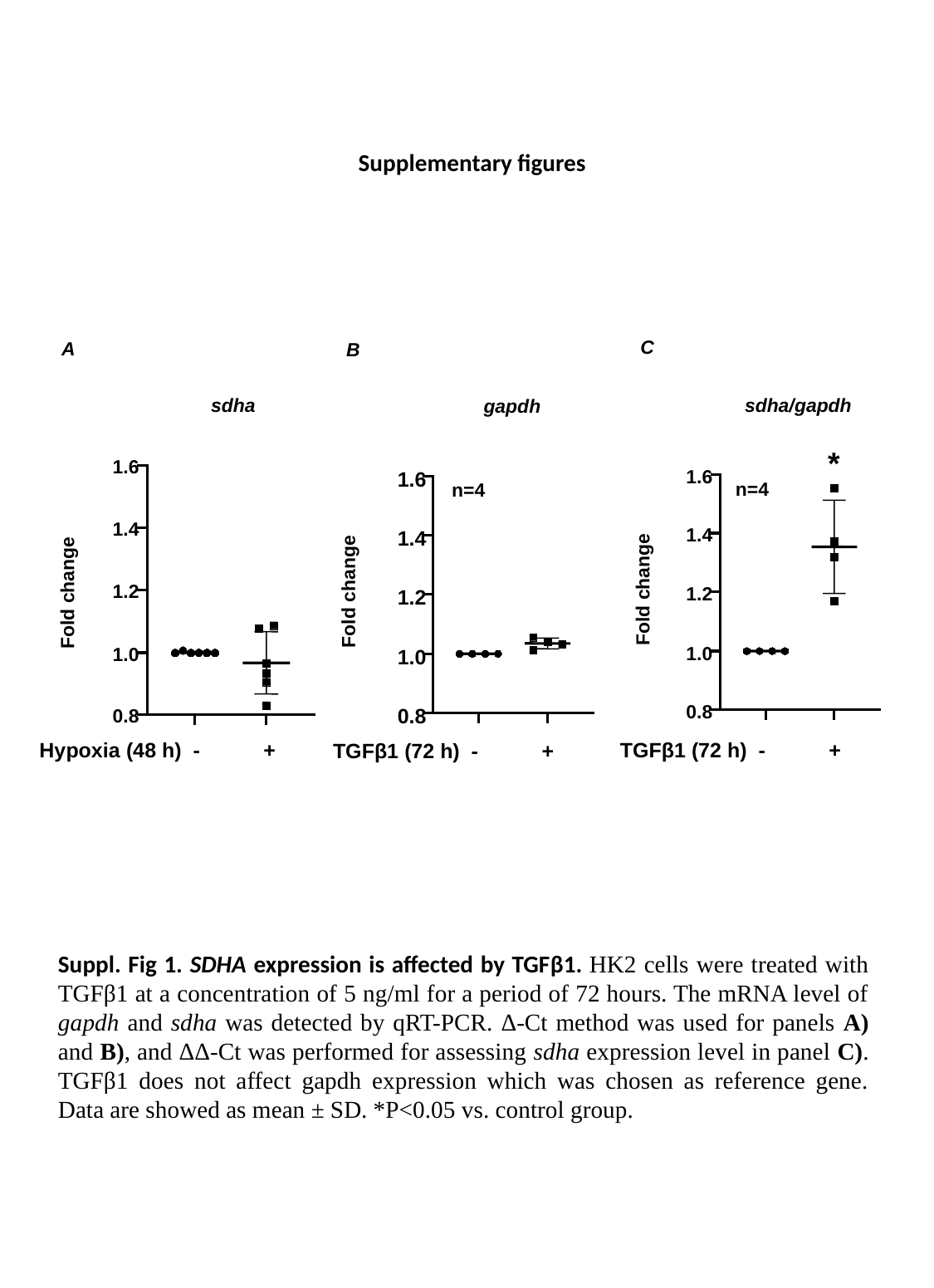

Supplementary figures
C
A
B
sdha
sdha/gapdh
gapdh
*
1.6
1.4
1.2
1.0
0.8
1.6
1.4
1.2
1.0
0.8
1.6
n=4
n=4
1.4
Fold change
Fold change
Fold change
1.2
1.0
0.8
 Hypoxia (48 h) - +
 TGFβ1 (72 h) - +
 TGFβ1 (72 h) - +
Suppl. Fig 1. SDHA expression is affected by TGFβ1. HK2 cells were treated with TGFβ1 at a concentration of 5 ng/ml for a period of 72 hours. The mRNA level of gapdh and sdha was detected by qRT-PCR. Δ-Ct method was used for panels A) and B), and ΔΔ-Ct was performed for assessing sdha expression level in panel C). TGFβ1 does not affect gapdh expression which was chosen as reference gene. Data are showed as mean ± SD. *P<0.05 vs. control group.

## Slide 2
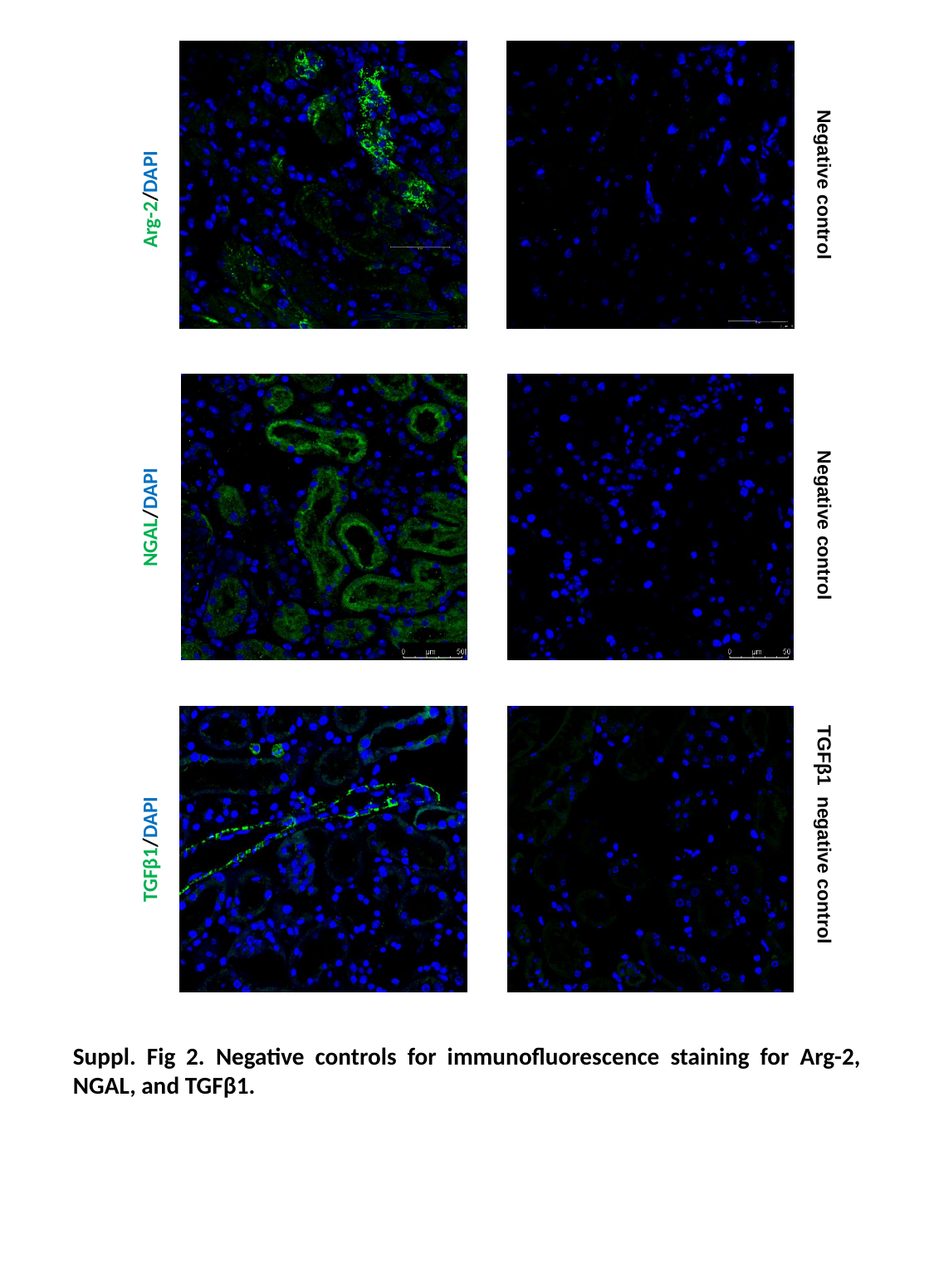

Negative control
Arg-2/DAPI
NGAL/DAPI
Negative control
TGFβ1 negative control
TGFβ1/DAPI
Suppl. Fig 2. Negative controls for immunofluorescence staining for Arg-2, NGAL, and TGFβ1.

## Slide 3
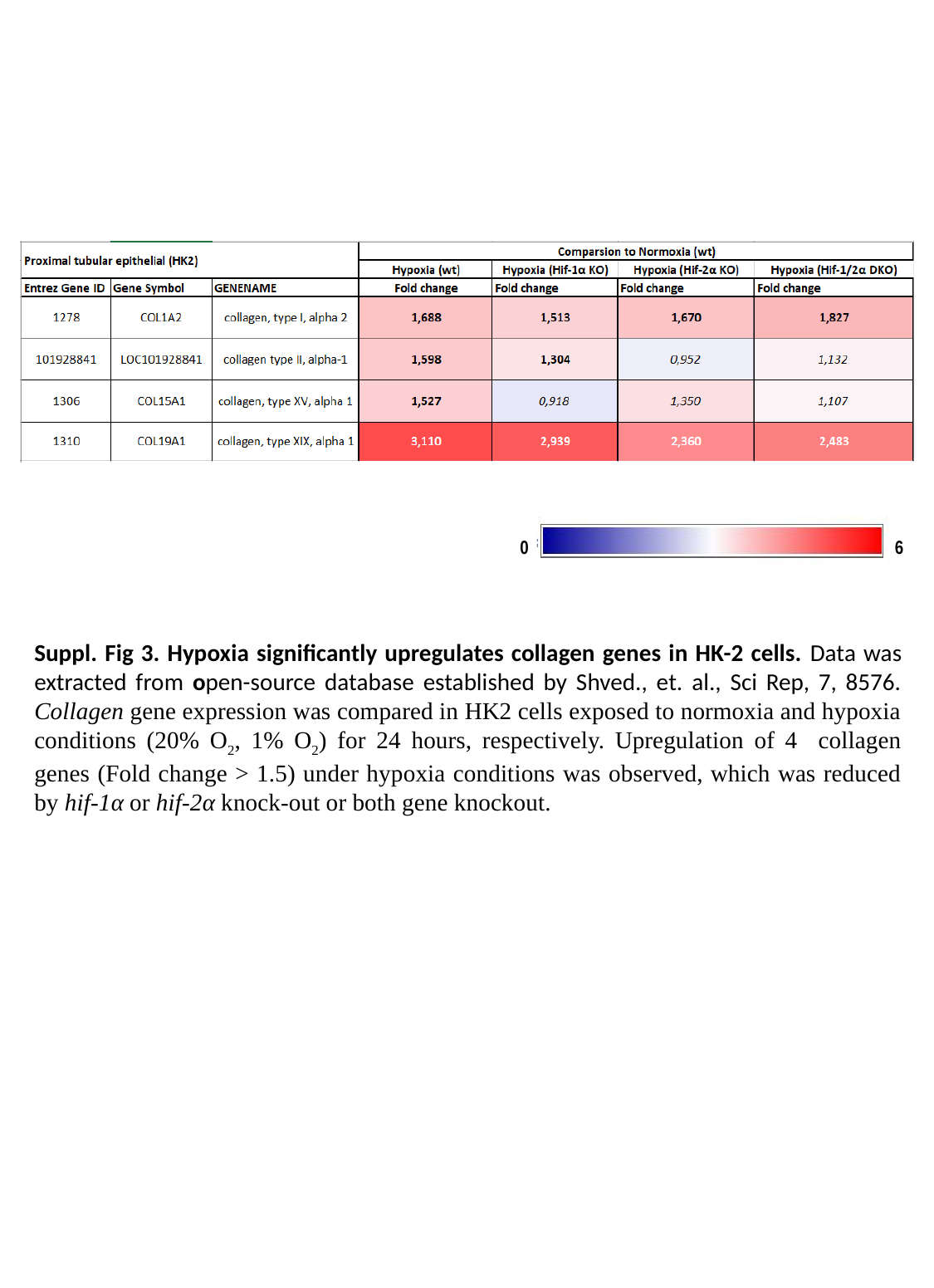

0
6
Suppl. Fig 3. Hypoxia significantly upregulates collagen genes in HK-2 cells. Data was extracted from open-source database established by Shved., et. al., Sci Rep, 7, 8576. Collagen gene expression was compared in HK2 cells exposed to normoxia and hypoxia conditions (20% O2, 1% O2) for 24 hours, respectively. Upregulation of 4 collagen genes (Fold change > 1.5) under hypoxia conditions was observed, which was reduced by hif-1α or hif-2α knock-out or both gene knockout.
