# Supplementary material for: Hypoxia Induces Renal Epithelial Injury and Activates Fibrotic Signaling Through Up-Regulation of Arginase-II
Source: Front Physiol. 2021 Nov 19;12:773719. doi: 10.3389/fphys.2021.773719 (PMC8640467; doi:10.3389/fphys.2021.773719)
Supplement: Supplementary file 2 [file Table_1.DOCX]

| **Antibody target** | **Dilution** |
| --- | --- |
| HIF1α | WB 1:1,000 |
| HIF2α | WB 1:1,000 |
| Arg-II (cell signaling, #55003) | WB 1:1,000; IF: 1:200 |
| β-actin | WB 1:10,000 |
| Tubulin | WB 1:15,000 |
| TGF-β1 | WB 1:1,000; IF:1:200 |
| NGAL | IF: 1:200 |
| IRDye 800-conjugated affinity purified goat anti-rabbit IgG | WB 1:5,000 |
| Alexa fluor 680-conjugated goat anti-mouse IgG | WB 1:5,000 |
| Alexa Fluor 488-conjugated goat anti-rabbit IgG (H+L) secondary Ab | IF 1:400 |

**Supplementary Table 1. Antibody dilutions used for immunoblotting and immunofluorescence**
